# Visual health screening by schoolteachers in remote communities of Peru: implementation research

**DOI:** 10.2471/BLT.15.163634

**Published:** 2016-06-21

**Authors:** Sergio Latorre-Arteaga, Diana Gil-González, Covadonga Bascarán, Richard Hurtado Núñez, María del Carmen Peral Morales, Guillermo Carrillo Orihuela

**Affiliations:** a Public Health Research Group, University of Alicante, Campus San Vicente del Raspeig, Ap. 99 CP 03080, San Vicente del Raspeig, Alicante, Spain.; b International Centre for Eye Health, London School of Hygiene & Tropical Medicine, London, England.; c Regional Educational Direction of Apurimac, Abancay, Peru.; d ONCE Foundation for Solidarity with Blind People in Latin America, Madrid, Spain.; e Universidad Peruana Los Andes, Lima, Peru.; Correspondence to Sergio Latorre-Arteaga (email: sergio.latorre@ua.es).

## Abstract

**Objective:**

To describe the adaptation and scaling-up of an intervention to improve the visual health of children in the Apurimac region, Peru.

**Methods:**

In a pilot screening programme in 2009–2010, 26 schoolteachers were trained to detect and refer visual acuity problems in schoolchildren in one district in Apurimac. To scale-up the intervention, lessons learnt from the pilot were used to design strategies for: (i) strengthening multisector partnerships; (ii) promoting the engagement and participation of teachers and (iii) increasing children’s attendance at referral eye clinics. Implementation began in February 2015 in two out of eight provinces of Apurimac, including hard-to-reach communities. We made an observational study of the processes and outcomes of adapting and scaling-up the intervention. Qualitative and quantitative analyses were made of data collected from March 2015 to January 2016 from programme documents, routine reports and structured evaluation questionnaires completed by teachers.

**Findings:**

Partnerships were expanded after sharing the results of the pilot phase. Training was completed by 355 teachers and directors in both provinces, belonging to 315 schools distributed in 24 districts. Teachers’ appraisal of the training achieved high positive scores. Outreach eye clinics and subsidies for glasses were provided for poorer families. Data from six districts showed that attendance at the eye clinic increased from 66% (45/68 children referred) in the pilot phase to 92% (237/259) in the implementation phase.

**Conclusion:**

Adaptation to the local context allowed the scaling-up of an intervention to improve visual health in children and enhanced the equity of the programme.

## Résumé

**Objectif:**

Décrire l'adaptation et l'extension d'une campagne visant à améliorer la santé visuelle des enfants dans la région d'Apurimac, au Pérou.

**Méthodes:**

Dans le cadre d'un programme pilote de dépistage mené dans un district de la région d'Apurimac en 2009–2010, 26 enseignants avaient été formés pour réaliser un dépistage de l'acuité visuelle chez les enfants scolarisés et orienter les enfants présentant des troubles visuels vers un centre de soins. Dans le but d'étendre cette campagne, les leçons tirées du programme pilote ont été exploitées pour élaborer des stratégies pour: (i) intensifier les partenariats multisectoriels; (ii) promouvoir l'implication et la participation des enseignants et (iii) augmenter le nombre d'enfants pris en charge dans un centre ophtalmologique. La mise en œuvre a débuté en février 2015, dans deux des huit provinces de la région d'Apurimac, notamment au sein de communautés difficiles à atteindre. Nous avons mené une étude observationnelle des activités d'adaptation et d'extension de la campagne et de leurs résultats. Des analyses qualitatives et quantitatives ont été réalisées à partir des données collectées entre mars 2015 et janvier 2016 dans la documentation du programme, les rapports de routine et les questionnaires structurés d'évaluation remplis par les enseignants.

**Résultats:**

Après la communication des résultats de la phase pilote, les partenariats se sont diversifiés. Une formation a été suivie par 355 enseignants et directeurs, dans les deux provinces; ce qui représente 315 écoles, réparties dans 24 districts. L'évaluation par les enseignants de la formation dispensée a été très positive. Un centre ophtalmologique de proximité ainsi que des aides financières pour l'achat de lunettes ont été mis en place pour les familles les plus pauvres. Les données obtenues dans six districts montrent que la prise en charge par un centre ophtalmologique est passée de 66% pendant la phase pilote (45 enfants sur les 68 orientés vers un centre de soins) à 92% pendant la phase de mise en œuvre (237 sur 259).

**Conclusion:**

L'adaptation au contexte local a permis d'étendre la campagne visant à améliorer la santé visuelle chez l'enfant et de renforcer la dimension d'équité du programme.

## Resumen

**Objetivo:**

Describir la adaptación y la ampliación de una intervención para mejorar la salud visual de niños de la región Apurímac, Perú.

**Métodos:**

En un programa piloto de examen realizado en 2009 y 2010, 26 profesores de escuela recibieron formación para detectar y tratar problemas de agudeza visual de los alumnos de un distrito de Apurímac. A fin de ampliar la intervención, las lecciones aprendidas en el piloto se utilizaron para diseñar estrategias para: (i) fortalecer las colaboraciones entre sectores; (ii) fomentar el compromiso y la participación de los profesores; y (iii) aumentar la asistencia de los niños a clínicas oftalmológicas de referencia. La implementación comenzó en febrero de 2015 en dos de ocho provincias de Apurímac, incluidas las comunidades de difícil acceso. Se utilizó un estudio de observación de los procesos y resultados de la adaptación y ampliación de la intervención. Se realizaron análisis cuantitativos y cualitativos de datos recopilados desde marzo de 2015 hasta enero de 2016 a partir de documentos del programa, informes rutinarios y cuestionarios de evaluación estructurados que los profesores habían rellenado.

**Resultados:**

Las colaboraciones aumentaron tras la publicación de los resultados de la fase piloto. Los que recibieron formación fueron 355 profesores y directores de ambas provincias, los cuales pertenecían a 315 escuelas distribuidas en 24 distritos. La evaluación de la formación por parte de los profesores obtuvo puntuaciones muy positivas. Las familias más pobres obtuvieron acceso a clínicas oftalmológicas a domicilio y ayudas para gafas. Los datos recopilados en seis distritos mostraron que la asistencia a clínicas oftalmológicas aumentó de un 66% (45/68 niños atendidos) durante la fase piloto a un 92% (237/259) durante la fase de implementación.

**Conclusión:**

La adaptación al contexto local permitió la ampliación de una intervención para mejorar la salud visual de los niños y aumentó la equidad del programa.

## ملخص

**الغرض:**

وصف أسلوب التكيّف وزيادة نطاق التدخل لتحسين الصحة البصرية للأطفال المقيمين في منطقة ابوريماك، بيرو.

**الطريقة:**

في برنامج الفحص الدليلي الذي أجري في الفترة من عام 2009 إلى عام 2010، تم تدريب المعلمين لاكتشاف مشكلات حدة البصر وإحالتها للعلاج في أطفال المدارس المقيمين في مقاطعة واحدة في منطقة ابوريماك. لزيادة نطاق التدخل، تمت الاستفادة من الدروس المستفادة الناتجة من البرنامج الدليلي لوضع الاستراتيجيات المخصصة لما يلي: (ا) تعزيز الشراكات في متعدد القطاعات؛ و(2) تشجيع انخراط ومشاركة المعلمين؛ و(3) زيادة تواجد الأطفال في عيادات العيون المحالين إليها. وبدأ التنفيذ في شهر فبراير من عام 2015 في مقاطعتين من بين ثمان مقاطعات في منطقة ابوريماك، بما في ذلك المجتمعات التي يكون من الصعب الوصول إليها. لقد قمنا بإجراء دراسة رصدية للعمليات والنتائج الخاصة بأسلوب تهيئة وزيادة نطاق التدخل. وتم إجراء مجموعة من التحاليل النوعية و الكمية للبيانات المجمعة من مارس من عام 2015 إلى يناير من عام 2016 من وثائق البرنامج، والتقارير الدورية، واستبيانات التقييم المنظمة التي يقوم المعلمين باستكمالها.

**النتائج:**

تم توسيع مدى الشراكات بعد مشاركة النتائج الخاصة بالمرحلة الدليلية. وتم استكمال التدريب بواسطة 355 من المعلمين والمدراء في كلتا المقاطعتين، والذين ينتمون إلى 315 مدرسة موزعة في 24 منطقة. ولقد حقق تقييم المعلمين للتدريب نتائج إيجابية مرتفعة. تم توفير عيادات العيون الميدانية والنظارات كوسائل دعم للعائلات الفقيرة. أظهرت البيانات الواردة من ست مناطق أن تواجد الأطفال في عيادات العيون قد زادت نسبته من 66% (45/68 من الأطفال المحالين للعلاج) في المرحلة الدليلية إلى 92% (237/259) في مرحلة التنفيذ.

**الاستنتاج:**

إن التكيّف مع السياق المحلي سمح بزيادة نطاق التدخل لتحسين الصحة البصرية في الأطفال وتحسين معايير المساواة للبرنامج.

## 摘要

**目的:**

为了描述一项干预措施的应用和规模扩展，以改善秘鲁阿普里马克大区儿童的视力健康。

**方法:**

在一项于 2009-2010 年间进行的试点筛查项目中，阿普里马克大区每个区对 26 位学校教师进行了培训，以检测和记录小学生视敏度问题。为了扩大干预措施的实施规模，从本次试点试验中得到的经验教训被应用到以下策略设计中：(i) 加强多部门合作；(ii) 提高教师的加入和参与，并且(iii) 提高转诊眼科诊所的儿童就诊率。项目于 2015 年 2 月在阿普里马克大区 8 个省中的 2 个省开始实施，包括难以到达的偏远社区。我们针对干预措施的应用和规模扩展的过程和结果进行了一项观察研究。我们对 2015 年 3 月到 2016 年 1 月期间从项目文件、例行报告和由教师完成的结构性评估问卷中收集的数据进行了定性和定量分析。

**结果:**

分享试点阶段取得的结果后，合作得到了进一步扩展。两个省份的 355 位教师和辅导员完成了培训，这些教师和辅导员来自 24 个区的 315 个学校。教师们的培训评估取得了很高的积极分数。同时，比较贫穷的家庭还获得了他们平常难以触及的眼科诊疗机会和眼镜补贴。来自 6 个区的数据显示，眼科诊所的就诊率从试点试验阶段的 66%（45/68 转诊儿童）提高至实施阶段的 92% (237/259)。

**结论:**

可通过适应当地背景扩大干预措施的规模，从而改善儿童的视力健康，并提高项目的公平性。

## Резюме

**Цель:**

Описать адаптацию мероприятий по улучшению зрения детей к местным условиям и наращивание их масштабов в регионе Апуримак, Перу.

**Методы:**

В ходе пилотной программы проверки, проводимой в 2009–2010 гг., 26 школьных учителей в одном из округов региона Апуримак прошли подготовку, нацеленную на обнаружение нарушений остроты зрения у школьников и направление их за специализированной помощью. С целью расширения этой программы приобретенный опыт использовали при разработке стратегий: (i) для укрепления партнерских связей между несколькими секторами, (ii) поощрения вовлеченности и участия учителей и (iii) увеличения посещаемости специализированных офтальмологических клиник детьми. Внедрение было начато в феврале 2015 года в двух из восьми провинций региона Апуримак, в том числе в труднодоступных областях. Авторами статьи было проведено наблюдательное исследование процессов и результатов адаптации этих мероприятий к местным условиям и наращивания их масштабов. Были проведены качественные и количественные анализы на основании данных, собранных в период с марта 2015 года по январь 2016 года из программных документов, плановых отчетов и структурированных оценочных анкет, заполненных учителями.

**Результаты:**

Партнерские связи расширились после обмена результатами экспериментального этапа. Подготовку прошли 355 учителей и директоров, работающих в 315 школах, расположенных в 24 районах обеих провинций. Была проведена оценка подготовки учителей, показавшая высокие положительные результаты. Были созданы офтальмологические клиники для поддержки малоимущего населения, а также выделены пособия на очки для более бедных семей. Данные из шести районов свидетельствовали об увеличении посещаемости офтальмологических клиник с 66% (направлено 45 из 68 детей) на экспериментальном этапе до 92% (237 из 259) на этапе внедрения.

**Вывод:**

Адаптация к местным условиям позволила увеличить масштабы мероприятий по улучшению зрения детей и способствовала равному распределению действия программы.

## Introduction

Worldwide there are 12 million children with visual impairment due to uncorrected refractive errors that can affect their learning development.^1^^,^^2^ Evidence has shown that visual deficits can be identified through visual acuity testing and that affordable treatments to correct vision can improve the quality of life of the population.^3^ Some studies suggest that screening children’s vision is beneficial.^4^^,^^5^ The inclusion of visual health in a school’s curriculum can contribute to the development of a healthy school environment, promote good vision habits and permit the detection of eye problems, facilitating the integration of boys and girls with visual disability into the classroom.^6^

Apurimac region in the Andes mountains, consists of eight provinces and 79 districts, and has remote villages with hard-to-reach populations living at over 3000 metres in altitude; limited infrastructures and transportation; and poverty affecting a large proportion of the population.^7^ Despite these challenges, Apurimac has an educational network that covers the entire region.^8^ Access to eye-care services is limited due to a shortage of health professionals, particularly in rural areas.^9^

In 2009–2010, a pilot school visual health programme was carried out in Abancay, the capital city of Apurimac region (situated in a district and province of the same name), by a local eye-care centre with the participation of teachers and local education authorities.^10^ The objective was to estimate the proportion of schoolchildren with uncorrected refractive errors and to assess the validity of vision screening performed by trained schoolteachers. The prevalence of uncorrected refractive errors in the pilot sample of 364 children was 6.2% (13 children aged 3–5 years) and 6.9% (11 children aged 6–11 years), consistent with other studies in the region.^11^ There was a complete concordance between the visual acuity independently measured by 21 trained teachers and by expert health personnel. The pilot project highlighted a neglected visual health problem, because most of the examined children had never previously received an eye examination. The study also confirmed that trained schoolteachers were a valid resource for the identification of vision deficits in schoolchildren in this context. 

Using lessons learnt from feedback from the pilot programme we planned a scaled-up implementation of the screening programme to start in February 2015, with the goal of gradually covering the whole region in the coming years. The aim of this study was to describe the adaptation and scaling-up of the intervention to improve the visual health of children in the Apurimac region of Peru.

## Methods

### Intervention

The intervention was delivered in three stages: (i) training of teachers in visual acuity measurement and referral; (ii) screening of students by teachers and referral of children with visual deficits to eye-care services; and (iii) examination and treatment of children in an eye-care unit.

#### Training

Educational authorities at the regional level incorporated the programme in two of the eight provinces of Apurimac region. The training was integrated into the planning meeting that the local educational authorities in each province have with school principals and teachers at the beginning of the school year. The participants in the training were preschool, primary- and secondary-school teachers, and school directors. At least one teacher was trained per school, preferably the school principal.

Optometrists and ophthalmic nurses from the local eye hospital conducted the training with technical support provided by a nongovernmental organization participating in the programme. Teachers learnt how to accurately measure the children’s visual acuity at 6 metres in both eyes and to make referrals according to preset criteria. No previous knowledge of visual health was required to participate in the programme.

Training consisted of one session of 4 hours and included three theory modules on basic eye anatomy and visual function, common vision disorders in children and measuring visual acuity, followed by a practical module. Teachers were given a copy of the didactic materials, which included guidelines for reporting screening results and communicating with parents. The vision screening kit, consisting of visual acuity tests (adapted to reading and pre-reading ages), an eye occluder and measuring tape, were also supplied to each teacher.

#### Screening and referral

The screening programme covered preschool (age 3–5 years), primary-school (age 6–12 years) and secondary-school (age 13–18 years) children. The programme encouraged teachers to start the vision screenings of the children in their schools shortly after completing the training. In a talk given by a trained teacher before conducting the screening, the parents were informed verbally of the risks and benefits of the screening and asked to give consent for their child’s participation. To ensure confidentiality of the data, teachers informed parents individually in writing about the child’s screening results. Each participant was given a number and only one programme officer was allowed access to the referral information. 

The criteria for referral to the local eye-care centre were visual acuity at 6 metres for one or both eyes < 6/9 (20/30) for preschool children and ≤ 6/9 (20/30) for elementary schoolchildren. The observation by the teacher of any eye abnormalities, such as a white cornea, was also a criterion for referral. For preschool children two teachers performed the visual acuity test when possible and repeated it if the results met the criteria for referral. Parents of children identified for referral were informed in writing.

Eye examinations of referred children from the same school were programmed together as a group with the corresponding school principal and scheduled according to the school agenda and availability of the local eye hospital, in coordination with the families. Referred children attended eye examinations accompanied by a parent or teacher. 

#### Eye examinations

The local eye-care centre in the city of Abancay offered the eye examination free of charge for referred children and to participant teachers who requested one, and provided the necessary health personnel, clinical equipment and transport to conduct the outreach campaigns.

Eye examinations were performed by optometrists or ophthalmic nurses and followed the clinical procedure described in the pilot phase.^10^ In preschool children, hyperopia 3.00 dioptres and astigmatism 1.50 dioptres were considered the reference for minimum optical correction according to American Association of Pediatric Ophthalmology and Strabismus guidelines.^12^ For children older than 5 years, hyperopia 1.50 dioptres, astigmatism 0.75 dioptres (or lower, if signs and symptoms were present) and myopia 1.00 dioptres (or lower, if it improved visual acuity appreciably) were indicated as minimum values to prescribe optical correction, according to the proposed guidelines.^13^ The final prescription for glasses was determined taking into account the child’s vision reduction, symmetry of refractive error between both eyes and binocular function improvements, according to age. Children presenting with other pathologies were subsequently referred to an ophthalmologist at the local eye hospital or for a full medical examination at the nearest available hospital.

### Programme improvements

Before planning the implementation phase, we held a feedback meeting in June 2010 with participants in the pilot programme. This meeting revealed key aspects of the programme that needed to be improved to achieve greater effectiveness and impact. These were: (i) strengthening partnerships to achieve leadership and financial sustainability; (ii) promoting teachers’ engagement and participation; and (iii) increasing the referral attendance rate to improve the programme’s equity and impact. We therefore designed strategies to address these factors when planning the implementation phase (Table 1).

**Table 1:** Outcomes, strategies and data sources used to examine implementation of the school visual health programme in Apurimac region, Peru, 2015–2016

Outcome	Implementation strategies	Data sources
Partnerships	Disseminate pilot study results Recruit multidisciplinary partners	Analysis of pilot study results^10^ Memoranda of understanding with partners, programme reports and other institutional agreements
Teacher engagement	Increase teachers’ access to training programme Revise training materials and implementation of virtual campus Provide official certification of the training course Provide visual examination and treatment for teachers	Data on number of teachers accessing the course Data on number of schools involved Data on number of official certificates awarded Teachers’ scores on evaluation questionnaire completed after training^a^
Referral attendance	Develop health education materials for families Provide logistic and financial support for families Provide outreach clinics in remote locations	Programme awareness documents and materials Programme data on subsidies Programme data on number of outreach clinics Programme data on referral attendance rate^b^ Proportion of child population reached by district^c^

^a^ Structured questionnaire, with answers graded from 0 to 4 (strongly disagree to strongly agree). Teachers’ scores from each section of the questionnaire were aggregated into mean and standard deviation scores.

^b^ Number of children attending eye clinic as a percentage of number of children referred.

^c^ Number of children screened as a percentage of child population aged 3–17 years, attending school or not, by district (based on census data for Peru).^8^

#### Partnerships

The organizations responsible for carrying out the programme were the local secondary eye hospital Fundación Medico Oftalmológica Sagrado Corazón de Jesús, Peru, and the nonprofit organization Global Health Vision-Entretodos Foundation, Spain, leading the initiative, with the support of the University of Alicante, Spain. The partnership included the regional education directorate of Apurimac, local and international social organizations and educational institutions committed to the prevention of blindness and supporting people with disabilities (Table 2). 

**Table 2:** Partnerships in the implementation phase of the school visual health programme in Apurimac region, Peru, 2015–2016

Partner	Roles and responsibilities
Nonprofit organization leading the programme^a^	Design and develop the visual health programme and vision screening in the child population Search for funding sources Contribute to the training of teachers and raising social awareness Promote partnerships Provide evidence and models based on good practices towards universal access to eye health
Local eye hospital^b^	Provide comprehensive vision and diagnostic examinations Monitor and validate vision screening carried out by teachers Register patients who receive treatment Refer complex cases to specialized care within health services Provide vision health services to teachers when required
Regional education authority^c^	Certificate the training of teachers by providing curricular accreditation Coordinate and assist teachers in the training Participate in the planning, monitoring and evaluation of the programme
Municipal institutions^d^	Provide institutional and economic support to guarantee access to diagnosis and treatment for children referred after school screening Facilitate infrastructure to the health team during travel Support dissemination of the programme and raising social awareness in the local community
Universities and educational institutions^e,f^	Provide programme management Provide monitoring and evaluation Generate, transfer and disseminate scientific knowledge Facilitate training and support for health professionals
International organization supporting the inclusion of people with disabilities^g^	Provide specific training and resources for health and education sector professionals in terms of a school environment where children with disabilities can participate fully Identify children with visual disability who are not attending classes and facilitate their integration into the school
Other facilitators^h^	Contribute to expanding the social network to strengthen the programme in different ways: Community organizations: logistic support, transport of supplies and donation management Vision health specialists: support in teacher training, collaboration in design of training materials

^a^ Global Health Vision-Fundación Entretodos, Zaragoza, Spain.

^b^ Fundación Médico Oftalmológico Sagrado Corazón de Jesús, Abancay, Peru.

^c^ Dirección Regional de Educación de Apurimac, Abancay, Peru.

^d^ Local authorities and rural health post in both provinces at the district level, Abancay and Chuquibambilla, Peru.

^e^ Universidad de Alicante, Alicante, Spain and Universidad Peruana Los Andes, Huancayo, Peru.

^f^ International Centre for Eye Health, London, United Kingdom of Great Britain and Northern Ireland.

^g^ Fundación ONCE para América Latina, Madrid, Spain.

^h^ Madre Coraje; Jerez de la Frontera, Spain, Club de Leones Lima “La Recoleta”, Lima, Peru, Escoles Solidaries and eye care specialists, Valencia, Spain, Asociación Mirada a Perú, Lima, Peru.

To strengthen the partnerships for long-term leadership and financial sustainability, we disseminated the evidence of the pilot results^10^ and contacted potential partners. This resulted in agreements with seven new local and international stakeholders as well as collaboration with other facilitators. Their backgrounds, roles and responsibilities are summarized in Table 2.

#### Teacher engagement

Based on the results obtained in the pilot intervention, we introduced several strategies to increase the number of teachers who received training and completed the programme, and to ensure better follow-up of referred children.

First, we adapted and shortened the teacher training session. Training in the pilot study was carried out in two 4-hour sessions on consecutive days. In a sample of 21 feedback questionnaires, teachers were slightly dissatisfied with the duration of the training.^10^ Therefore, in the implementation phase we reduced the time for theoretical content by providing additional printed materials and focusing on practice (the visual acuity test, data collection and informing parents) in a single 4-hour session. Second, in partnership with the regional education authorities, official certification for the training course was obtained. Third, the teaching materials were revised with the contributions of visual health and education specialists from the partnership. Fourth, in addition to the paper-based system for training and recording data, an online virtual campus was designed and presented to teachers to facilitate their access to teaching materials, reporting of results and referral and follow-up of children identified with vision problems. Fifth, education resources on school vision health were made available through the virtual campus, including recommendations for integration of visually impaired children into the school (defined by conventional standards).^14^ Finally, with the aim of improving the engagement of teachers, as part of the training session we offered all teachers a full eye examination and provided them with glasses if required.

#### Referral attendance

Several strategies were put in place to improve the relatively low attendance rate at the eye centre observed in the pilot programme (66%; 45 out of 68 children referred), and to improve equity of access to eye-care services, by including rural teachers and communities in the programme. The health team from the local eye-care centre in the pilot were also responsible for the implementation of the scaling-up.

First, we increased awareness about visual health among the local community and families by providing the schools with posters and information leaflets which they distributed before the screening started.

Second, to overcome the barriers discussed in the pilot, we introduced logistic support for families in the rural areas, initially by covering the cost of transport to the eye hospital for referred children. Due to the number of children referred by teachers from remote areas – where travelling to and from eye examinations by public transport cannot be achieved in a single day – we found that organizing an outreach clinic was a more efficient and patient-centred way to offer the examination and treatment. Deployment of a mobile unit from the local eye hospital to remote municipalities was arranged, coordinated through links with municipalities and the national network of rural health posts that supported the implementation of the programme.

Third, financial subsidies for patients who could not afford the cost of glasses, medical treatment or transport to the eye hospital were offered from cooperation funds and crowdfunding through the local eye hospital. The programme established three principles for the correction of refractive errors for the children identified through the screening: (i) no child with vision problems should lack treatment due to financial reasons; (ii) families can contribute fully or partially to the cost of the treatments; and (iii) all service providers involved in the school visual health programme participate on a nonprofit basis. The cost of quality glasses sourced from local providers, including durable, plastic lenses, cutting, assembling and transport, was estimated at 15 United States dollars (US$) each. Payment options for families were the full cost of the treatment, a partial contribution or no contribution; the payment option for each child was selected by the family during the examination according to self-reported financial status or, in the absence of parents, following teacher recommendations.

### Implementation

Implementation of the scaled-up intervention, which is still in progress, began in February 2015 in Abancay and Grau, two of eight provinces in Apurimac region. Training sessions were carried out in institutional facilities in the capital cities of these provinces, Abancay and Chuquibambilla respectively, in February and March 2015.

All partners provided the financial, human and logistic resources needed to implement the scaling-up programme. A collective crowdfunding campaign generated funds and optical and ophthalmic equipment were donated to the programme. Local authorities at the district level assisted by accommodating health staff and providing a suitable location for the eye clinic, i.e. a rural health post or a classroom with suitable lighting and a room at least 5 metres long.

### Outcomes and data sources

We considered three outcomes of the implementation process, corresponding to the three areas targeted for scaling-up the programme: (i) partnerships; (ii) teacher engagement; and (iii) referral attendance. Table 1 shows the sources and types of data collected under each outcome; these included regular field visits, routine programme records, programme documents and reports, and structured evaluation questionnaires completed by teachers. Qualitative and quantitative analyses were made of data collected for the period of implementation from March 2015 to January 2016. We also present data on the causes of decreased visual acuity collected from records of a sample of 63 children attending the local eye-care centre.

## Results

The collaboration with the regional education directorate of Apurimac resulted in the intervention being included in the official school agenda in Apurimac.

### Teacher engagement

The number of teachers and directors completing training were 355 in both provinces, belonging to 315 schools distributed in 24 districts, compared with 21 teachers from one district in the pilot intervention. Overall the teachers agreed or strongly agreed that the training session was useful, gave them the skills needed for screening and was well delivered. 

Due to the adaptations needed to include a one-hour practice and the delivery of visual health materials for schools during training sessions, teacher participants were distributed into groups. The same process was followed for all groups, except that questionnaires were collected from only one in four groups due to the limited time available. The mean scores of the teachers’ assessment of the training (from 0 to 4) for each item in a sample of 84 questionnaires collected were as follows: “The training contents are useful for my performance as a teacher” 3.7 (standard deviation, SD: 0.5); “I understand the material taught in the training session and I feel capable of performing vision screening test” 3.7 (SD: 0.6); “The structure of the sessions has been adequate” 3.5 (SD: 0.7). The single 4-hour session applied in the implementation was better received by teachers and they showed good confidence in the acquired knowledge during training.

The estimated cost of training and equipping one teacher to perform the vision screening, calculated considering the resources procured from local providers, was US$ 5.

### Referral attendance

The data on screening outcomes and coverage, disaggregated by district, obtained in the implementation phase compared with the pilot phase are shown in Table 3. Between March 2015 and January 2016, 74 teachers from 6 districts screened a total of 1522 children and 259 (17%) met the criteria for referral. The proportion of the total child population aged 3–17 years who were screened ranged from 4% (625/17 862) in Abancay district to 24% (364/1516) in Gamarra district. From the referred children, 237 (92%) received a comprehensive eye examination. Except in Abancay, the majority of children came from remote rural areas (Table 3).

**Table 3:** Screening data and outcomes from the pilot and implementation phases of the school visual health programme by district of Apurimac region, Peru, 2015–2016

Phase and district	Children’s characteristics^a^				Screening outcomes				
	Population aged 3–17 years^a^	Living in rural areas, no. (%)^b^	Completing primary school, no. (%)^c^		Teachers trained	Teachers reporting, no.	Children screened, no. (%)^d^	Children referred, no.	Children examined, no. (%)^e^
**Pilot phase** Abancay	17 862	1 964 (11)	14 647 (82)		25	21	387 (2)	68	45 (66)
**Implementation phase^f^**	25 081	17 005 (68)	17 556 (70)		355^g^	74	1 522 (6)	259	237 (92)
Abancay	17 862	1 964 (11)	14 647 (82)		47	29	625 (4)	85	80 (94)
Circa	824	709 (86)	626 (76)		18	10	108 (13)	13	11 (85)
Huanipaca	1 720	1 341 (78)	1 135 (66)		22	7	157 (9)	18	13 (72)
Lambrama	1 677	1 191 (71)	1 325 (79)		17	8	144 (9)	26	22 (85)
Pichirhua	1 482	1 550 (87)	845 (57)		22	6	124 (8)	19	16 (84)
Gamarra	1 516	1 359 (90)	894 (59)		38	14	364 (24)	98	95 (97)

^a^ Total census population aged 3–17 years, attending school or not, by district. Data sourced from Instituto Nacional de Estadística e Informática, 2010.^8^

^b^ Number and percentage of children aged 3–17 years living in rural areas.^8^

^c^ Number and percentage of children completing primary education at the age of 12–13 years.^8^

^d^ Number of children screened as a percentage of child population aged 3–17 years.

^e^ Number of children examined at eye centre or outreach clinics as a percentage of children referred.

^f^ Based on data collected from March 2015 to January 2016.

^g^ Numbers of teachers trained by district do not total 355 as there were teachers trained from other districts that did not report data during the study period.

Over three quarters of the children examined (147; 62%) required treatment for refractive errors, giving a prevalence of uncorrected refractive error in this sample of children screened of 10%. Regarding financial contributions towards the cost of treatments, 53 (36%) of the 147 families were able to pay fully for glasses, 30 (20%) were able to pay partially and 64 (44%) required full subsidy.

### Vision problems detected

The main cause of decreased visual acuity in the 63 children examined at the local eye hospital was refractive error (60 children; 95%) (Table 4) and the most prevalent refractive error was astigmatism (47 children; 75%) with a mean value of 2.25 dioptres (SD: 1.77). Amblyopia, defined as decreased vision in one or both eyes even after best optical correction, was found in 12 children (19%). Conjunctivitis was the cause of poor vision in 9 (14%) children. Other eye diseases identified were corneal trauma and retinal disorder in 3 children (5%).

**Table 4:** Causes of decreased visual acuity in schoolchildren referred by teachers in the implementation phase of the school visual health programme in Apurimac region, Peru, 2015–2016

Cause	Children diagnosed, no. (%) (*n* = 63^a^)	Mean (SD) refractive error, dioptres
**Refractive error**	60 (95)	–
Astigmatism	47 (75)	–2.25 (1.77)
Myopia	16 (27)	–1.50 (1.11)
Hyperopia	13 (21)	1.00 (0. 73)
**Amblyopia**	12 (19)	N/A
**Conjunctivitis**	9 (14)	N/A
**Other causes**	3 (5)	N/A

N/A: not applicable; SD: standard deviation.

^a^ Number of children attending the local eye hospital, (numbers do not total 63 as children could have more than one diagnosis).

## Discussion

This paper presents the implementation strategy and processes of a school visual health programme with the participation of trained teachers and the involvement of a multisector partnership. Our results show improvements in the scope and effectiveness of the intervention compared with the pilot programme. The attendance rate at referral visits increased by 39% (from 66% to 92%) in the scaled-up intervention. This was achieved by offering support to poor communities from remote areas to ensure the delivery of a more equitable programme.

The main cause of decreased visual acuity in the children examined was refractive error (95%). The prevalence of uncorrected refractive errors in this sample (10%) was higher than in the pilot programme (6.6%) and higher than presented in another study in the same area (4.6%).^11^

The pilot study was carried out mostly in an urban population, in contrast with the implementation that also covered hard-to-reach areas. By targeting hard-to-reach populations this programme specifically included children who were more likely to be deprived of eye-care services and therefore more likely to have an uncorrected refractive error. Similar findings about a higher rate of uncorrected refractive errors in rural populations was reported by a school screening programme in India.^16^

We also confirmed that astigmatism was the most common refractive error in the Andean region of Peru.^11^ Our findings show that 19% of children with uncorrected refractive errors presented with amblyopia. In a study from Chile, 6.5% of 1285 schoolchildren with decreased visual acuity had amblyopia.^15^ Although the prevalence of refractive errors reflected the entire sample, data on causes for decreased visual acuity were limited to the children attending the local eye-care centre (Table 4). There are now arrangements in place to ensure that disaggregated data collection for outcomes are recorded and completed by age and area (rural or urban) for all children examined, with the appointment of a programme technician dedicated to this. This support will allow future analysis of the impact of the programme on schoolchildren’s visual function. For instance, further analyses will determine the most appropriate ages to conduct the screening and the optimum screening intervals.^13^

While programme coverage is increasing gradually in both the provinces studied, unexpected external and contextual factors affected the implementation of the programme within the planned time frame. During the scale-up implementation the region was affected by a general strike shortly after the training, which paralysed school activity, temporarily affecting the screening programme in most districts. This had repercussions on the number of teachers reporting data during this period. Additionally, some rural schools were staffed by only one teacher, who had little time available to perform the screenings or to facilitate the attendance of the referred children at the eye-care centre via communications with parents and the local eye hospital. Lack of electricity or access to telephone and Internet in these rural educational centres made communication and programme follow-up difficult. Consequently, teachers from these areas could not take advantage of the virtual campus to engage with the discussion forum and input their data. Data from paper records were collected from all the teachers included in the study.

As a result of these contextual factors only 74 teachers from 6 districts had reported screening results by January 2016. In these districts a total of 6% of the child population was reached by the programme. Due to the high rates of attendance for examinations among the referred children, including those in remote areas, in one district with a higher participation of teachers (Gamarra), the programme reached up to 24% of the total child population, despite its having a higher percentage of rural population and a lower school attendance rate than other districts.

Outreach clinics proved to be a feasible solution to reach children in distant locations and were organized despite not being originally planned or budgeted for. As a result, teachers and families with children referred had to walk distances of up to 5 hours to reach the mobile eye unit. Going forward, additional resources will be put in place to further support children in remote locations.

Another potential barrier to the scaling-up of the programme is the fact that only one provider was responsible for examination and treatment of children in the eye-care centre and the mobile clinic. Although optometry services at the local eye-care centre are being strengthened by providing training and clinical equipment, we need to involve other providers to address an increasing demand for services.

Strategies adopted to prepare the programme for long-term sustainability succeeded in increasing the number of local and international partners. The involvement of the educational authorities was instrumental in making the vision screening programme official for the region. The programme does not depend solely on any one of the partners, thus decreasing the risk if any of them ceased collaboration. For instance, the programme was maintained through changes in leadership of the regional education institutions. Financial contributions to support the programme are shared across the partners, including teacher and child participants.

The generalizability of these findings is influenced by the specific context in which the programme was implemented. The success of these interventions in other locations may depend on adaptation to the local geographical and socioeconomic needs. However, there is evidence in the literature of the success of screening by teachers in other low- and middle-income settings.^6^

To conclude, adaptation to the local context allowed the development of an effective scale-up intervention to improve visual health in children that could help to improve educational outcomes.^17^ Data collection to evaluate the overall outcome of the intervention is still on-going and requires longer term programme implementation. Going forward, the collaboration between the stakeholders should focus on the programme’s objective of improving visual health in Apurimac and integration of visually impaired children, including those children who may not be attending school. Further implementation research could contribute to identifying key factors that can improve the effectiveness of school visual health programmes in different contexts.
